# Immune Checkpoint Inhibitor–Associated Hemophagocytic Lymphohistiocytosis: A Systematic Review

**DOI:** 10.1177/11795549261489492

**Published:** 2026-09-11

**Authors:** Abdulrahman F. Al-Mashdali, Fatihelmgib Mohamed, Marwa Osman, Mujahid O. Abdelraof, Mohammed Abdulgayoom, Shehab F. Mohamed

**Affiliations:** 1 Department of Hematology, National Center for Cancer Care and Research, Hamad Medical Corporation, Doha, Qatar; 2 Blue Nile National Institute for Communicable Disease, Gezira University, Sudan; 3 Faculty of Medicine, Shendi University, Sudan

**Keywords:** immune checkpoint inhibitors, hemophagocytic lymphohistiocytosis, macrophage activation syndrome, immune-related adverse events, systematic review

## Abstract

**Background:**

Hemophagocytic lymphohistiocytosis (HLH) is a rare, life-threatening hyperinflammatory toxicity increasingly reported with immune checkpoint inhibitors (ICIs). We systematically reviewed published cases of ICI-associated HLH to characterize presentation, diagnostic patterns, treatment, outcomes, and rechallenge experience.

**Methods:**

PubMed/MEDLINE, Scopus, and Web of Science were searched from inception to 10 March 2026 according to a prior protocol. English-language case reports and case series reporting ICI-associated HLH or macrophage activation syndrome with extractable patient-level data were included. Data were synthesized descriptively. The review was retrospectively registered with the Open Science Framework (OSF; DOI: 10.17605/OSF.IO/6ARJC).

**Results:**

Ninety-six patients from 78 reports were included: 65 case reports and 13 case series. Median age was 60.0 years (IQR, 42.5–72.0; range, 2–86), and 53 patients (55.2%) were male. The most common malignancies were melanoma (27.1%), lung cancer (24.0%), breast cancer (9.4%), and kidney cancer (8.3%). Among reported cases, PD-1 blockade was the most common ICI exposure (60/96, 62.5%), followed by mixed-class regimens (20/96, 20.8%). HLH occurred after a median of 2 ICI cycles and 30 days from ICI initiation.

Diagnostic ascertainment was heterogeneous: combined criteria were used in 31/96 patients (32.3%), HLH-2004 criteria in 27/96 (28.1%), HScore in 23/96 (24.0%), and clinician diagnosis in 15/96 (15.6%). Fever occurred in 94/96 patients (97.9%), cytopenias in 91/93 (97.8%), and ferritin was markedly elevated where reported, with a median of 10,450 ng/mL. Corticosteroids were administered in 96/96 patients (100%), whereas etoposide was used in 19/96 (19.8%), IL-6 blockade in 21/96 (21.9%), and IL-1 blockade in 10/96 (10.4%). Clinical improvement or resolution occurred in 78/94 patients (83.0%). Death occurred in 24/95 patients (25.3%). ICI rechallenge was reported in 7 patients, with recurrent HLH in 3.

**Conclusion:**

ICI-associated HLH should be considered early in ICI-treated patients presenting with fever, cytopenias, and marked hyperferritinemia. Published cases show heterogeneous diagnostic ascertainment, variable treatment escalation beyond corticosteroids, and substantial mortality, underscoring the need for standardized diagnostic workup, early multidisciplinary management, and cautious individualized decisions regarding ICI rechallenge.

## Introduction

Immune checkpoint inhibitors (ICIs) have transformed oncological care since ipilimumab’s approval for metastatic melanoma in 2011,^1^ with agents targeting CTLA-4, PD-1, and PD-L1 releasing physiological brakes on T-cell activation to restore antitumor immune surveillance.^2,3^ As of 2024, eleven ICI agents are FDA-approved across more than 43 oncological indications, with PD-1/PD-L1 inhibitors now standard-of-care in non-small cell lung cancer, melanoma, urothelial carcinoma, renal cell carcinoma, hepatocellular carcinoma, and hematological malignancies, increasingly as combination regimens targeting multiple checkpoints simultaneously.^4^ However, the same immune disinhibition underlying ICI efficacy predisposes patients to immune-related adverse events (irAEs) — the principal clinical limitation of checkpoint immunotherapy — which develop in over half of treated patients within the first year, with grade 3–4 events in up to 30% of those receiving dual checkpoint blockade.^5,6^ While common irAEs (dermatitis, thyroiditis, colitis, pneumonitis) are well characterized with established management guidelines, rare and severe toxicities remain incompletely understood and underreported,^5^ particularly hematological irAEs — including immune thrombocytopenia, autoimmune hemolytic anemia, aplastic anemia, and acquired hemophilia — which carry disproportionate morbidity relative to their incidence and remain a clinically underappreciated dimension of ICI toxicity.^7^

At the most severe end of this hematological spectrum lies hemophagocytic lymphohistiocytosis/macrophage activation syndrome (HLH/MAS), a life-threatening hyperinflammatory syndrome driven by pathological activation of macrophages and cytotoxic T lymphocytes, sustained IFN-γ overproduction, and progressive multiorgan failure.^1^ Clinically, HLH is characterized by persistent fever, cytopenias, hyperferritinemia, hepatosplenomegaly, coagulopathy, and — where assessed — elevated soluble CD25 and reduced NK-cell activity. The syndrome carries intrinsically high mortality regardless of etiology, and its recognition in the ICI context is compounded by substantial diagnostic complexity, as its clinical and laboratory features overlap extensively with malignancy progression, sepsis, and other concurrent irAEs in oncology patients.^2^ ICI-associated HLH was first described in 2017, and cases have since been reported with increasing frequency across tumor types and ICI classes, consistent with disproportionality signals identified in the WHO global pharmacovigilance database VigiBase, where HLH was reported seven times more frequently with ICI therapy than with other drug classes and three times more frequently than with other antineoplastic agents.^3^

Despite this emerging recognition, critical gaps persist across all domains of clinical knowledge. The existing published literature on ICI-associated HLH consists exclusively of case reports and small case series, with the largest prior reviews encompassing fewer than 52 patients and limited to earlier time periods,^4,5^ precluding a comprehensive and current characterization of the syndrome’s phenotype, treatment patterns, and outcomes. Neither the HLH-2004 diagnostic criteria — originally developed and validated in the pediatric primary HLH setting — nor the HScore — derived from a mixed adult reactive HLH population that did not include ICI-treated oncology patients — has been validated for use in this clinical context, and diagnostic ascertainment across the published literature has been profoundly heterogeneous.^6^ Management remains entirely empirical: no prospective trials, controlled studies, or comparative treatment analyses exist, and the roles of specific escalation strategies — including etoposide, IL-6 blockade, and IL-1 blockade — are informed only by uncontrolled observations from isolated cases.^4,5^

We conducted a systematic review and pooled patient-level analysis of all published cases of ICI-associated HLH/MAS, with the aim of providing the most comprehensive and current characterization of this rare but potentially fatal immune-related toxicity. Specifically, this review sought to characterize the clinical phenotype, diagnostic landscape, and laboratory features of ICI-associated HLH across all published cases; describe the spectrum and frequency of treatment approaches employed; determine clinical outcomes including treatment response, relapse, and mortality; examine concurrent factors including infection, malignancy progression, and co-occurring irAEs; and identify the critical gaps in diagnostic standardization, treatment evidence, and rechallenge safety that must be prioritized in future prospective research and guideline development.

## Methods

### Study Design and Reporting

This systematic review was conducted according to an internally developed prior protocol finalized before study screening and data extraction. The protocol specified the research question, eligibility criteria, information sources, screening process, data extraction domains, quality assessment approach, and planned descriptive synthesis. The protocol was not prospectively registered in PROSPERO. Following completion of the review, the protocol and review methodology were retrospectively registered on the Open Science Framework (OSF; DOI: 10.17605/OSF.IO/6ARJC) to provide a transparent, time-stamped record of the review methods. The review was designed as a descriptive systematic review using patient-level data extracted from case reports and case series, rather than a comparative meta-analysis. Reporting followed the PRISMA 2020 statement where applicable.^7^

### Information Sources and Search Strategy

A systematic literature search was conducted in three electronic databases: PubMed/MEDLINE, Scopus, and Web of Science. Databases were searched from inception to 10 March 2026 in all databases. The search strategy combined terms related to immune checkpoint inhibitors and HLH/macrophage activation syndrome, including: “immune checkpoint inhibitor,” “checkpoint blockade,” “PD-1,” “PD-L1,” “CTLA-4,” “pembrolizumab,” “nivolumab,” “ipilimumab,” “atezolizumab,” “durvalumab,” “avelumab,” “hemophagocytic lymphohistiocytosis,” “haemophagocytic lymphohistiocytosis,” “HLH,” “macrophage activation syndrome,” and “MAS.” The search syntax was adapted for each database. Only English-language articles were included. Reference lists of included articles and relevant reviews were manually screened to identify additional eligible reports. The complete database-specific search strategies are provided in Supplementary Table 1.

### Eligibility Criteria

Studies were eligible if they reported at least one patient with immune checkpoint inhibitor–associated hemophagocytic lymphohistiocytosis or macrophage activation syndrome. Eligible study designs included case reports and case series. Reports were included regardless of patient age, sex, cancer type, ICI agent, ICI class, ICI regimen, or treatment line.

Cases were eligible when HLH/MAS was diagnosed by the reporting authors using HLH-2004 criteria, HScore, a combined diagnostic approach, or clinician diagnosis supported by compatible clinical and laboratory findings.^2,6^ Reports were excluded if they did not describe ICI exposure, did not report HLH/MAS, lacked extractable patient-level data, represented non-human studies, or consisted of reviews, editorials, commentaries, or pharmacovigilance/database analyses without sufficient individual-patient clinical detail. Letters and correspondence were eligible if they reported extractable individual-patient data. Conference abstracts were excluded unless sufficient individual-patient clinical data were available for extraction.

### Study Selection

All retrieved records were imported into reference-management software (Zotero), and duplicates were removed before screening. Titles and abstracts were screened independently by two reviewers for eligibility, followed by independent full-text review of potentially relevant articles by the same two reviewers. Disagreements at any stage of screening were resolved through discussion and consensus. If consensus could not be reached, a third reviewer resolved the conflict. Reasons for exclusion at the full-text stage were recorded and summarized in the PRISMA flow diagram.

### Data Collection Process, Data Items, and Data Handling

Data were extracted using a standardized data collection form developed for this review. Data extraction was performed independently by two reviewers, and discrepancies were resolved by discussion and consensus. If agreement could not be reached, a third reviewer resolved the conflict. For publications combining an original case report with an accompanying literature review, only the authors’ own reported case(s) were extracted for the pooled analysis; the review component of such articles was used exclusively to identify additional candidate references for screening, and was not itself a source of patient-level data. Extracted variables included publication characteristics, patient demographics, primary malignancy or underlying disease, cancer stage, ICI class and agent, regimen type, treatment line, timing of HLH onset, diagnostic approach, HLH clinical and laboratory features, reported infectious and malignant co-factors, concurrent immune-related adverse events, treatment strategies, supportive care, organ-support requirements, clinical response, relapse, mortality, cause of death, ICI rechallenge, and HLH recurrence after rechallenge.

Primary malignancy was coded at the organ or disease level only, such as lung, melanoma, breast, kidney, stomach, bladder, cervix, and liver, without detailed histologic subclassification. Cancer stage was coded as stage 1, 2, 3, or 4 when applicable and reported. Extracted data were cleaned before analysis using predefined coding rules. “NR” was treated as not reported and was not imputed. “NA” was treated as not applicable and was excluded from applicable-case denominators when appropriate. Continuous variables were summarized only when extractable numeric values were available, whereas values reported only as ranges, decades, or non-specific descriptors were excluded from continuous-variable summaries. Categorical variables were harmonized to reduce variation in terminology, including ICI agent names, ICI class, malignancy type, stage, treatment category, and outcome classification. The primary outcomes were clinical response, mortality, and HLH recurrence after ICI rechallenge. Secondary outcomes included timing of HLH onset, diagnostic approach, HLH clinical and laboratory features, reported infectious or malignancy-related co-factors, ICI discontinuation, corticosteroid and adjunctive immunomodulatory therapy, ICU admission, organ-support requirements, relapse, and recorded cause of death.

### Quality Assessment

The methodological quality of included case reports and case series was assessed using the framework proposed by Murad et al, which evaluates case-based evidence across the domains of selection, ascertainment, causality, and reporting.^8^ Quality assessment was performed independently by two reviewers, with disagreements resolved by discussion and consensus. If consensus could not be reached, a third reviewer resolved the conflict. The appraisal was used to characterize the completeness and reliability of the included evidence and was not used as the sole basis for excluding eligible reports.

Overall study-level risk of bias was categorized using a domain-threshold approach: studies fulfilling ≥4 of the 5 domains (Q1–Q5) were classified as overall ‘Low Concern,’ whereas those with ≥2 unclear or unfulfilled domains were categorized as ‘Moderate Concern.’ Under this framework, studies demonstrate robust reporting quality and clinical fidelity across domains Q2 through Q5 achieved an overall ‘Low Concern’ rating despite the inherent selection uncertainty in Q1.

### Data Synthesis and Statistical Analysis

Analyses were descriptive. Categorical variables were summarized as frequencies and percentages using the total cohort denominator, available-case denominator, or applicable-case denominator, as appropriate and explicitly stated. Continuous variables were summarized using median, interquartile range, and range. No formal comparative meta-analysis was performed because the included studies were uncontrolled case reports and case series with heterogeneous reporting. Therefore, pooled findings were interpreted as descriptive summaries of published cases, not as estimates of incidence, prevalence, comparative risk, or treatment effect.

## Results

### Search Results

**Figure 1.**
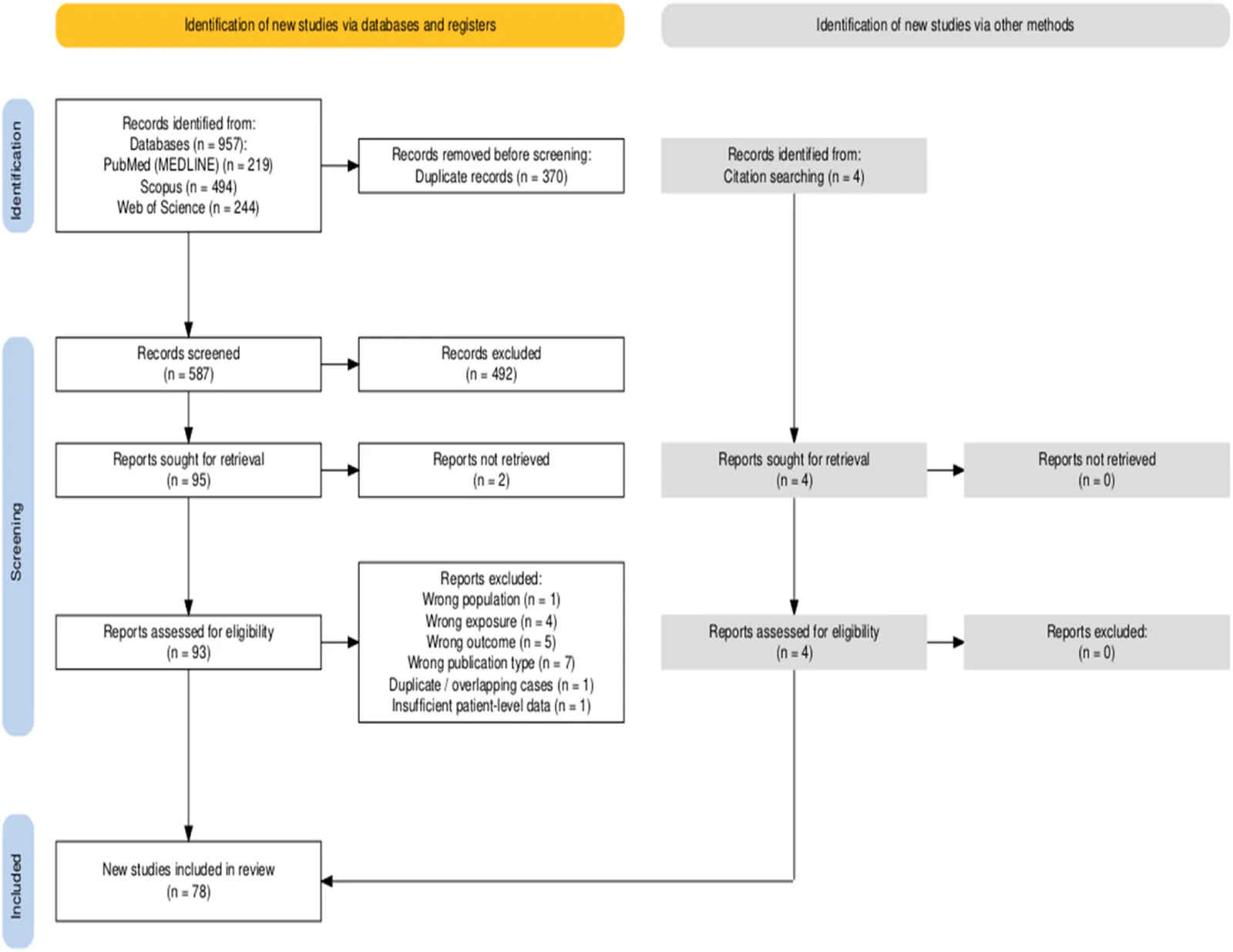
PRISMA flow diagram of study selection

A comprehensive literature search was conducted across PubMed/MEDLINE, Scopus, and Web of Science. The initial search retrieved 957 records; following the removal of 370 duplicates, 587 unique records underwent title and abstract screening. Of these, 492 were excluded for not meeting predefined inclusion criteria, and the full texts of the remaining 95 records were assessed for eligibility. An additional 4 records were identified through manual citation searching, yielding 99 full-text articles reviewed in total. After exclusion of 21 reports during full-text review, 78 studies published between 2017 and 2026 were ultimately included, comprising 96 patients who developed HLH following ICI therapy. The study selection process is detailed in the PRISMA flow diagram (Figure 1).

### Quality Assessment

Domain-level ratings for all 78 included studies are presented in Supplementary Table 1. Using the Murad tool, most included reports demonstrated adequate ascertainment of exposure and outcome, with clear documentation of ICI exposure, definitive HLH diagnoses, and sufficient clinical granularity to support investigator inference; a plausible temporal relationship between ICI exposure and HLH onset was established in the majority of cases. However, the selection domain was rated “Unclear” or “No” for the large majority of studies, reflecting the inherent limitations of case report/case series methodology, wherein cases are identified and reported at the discretion of the treating clinicians rather than through systematic or consecutive ascertainment. This pattern raises the possibility that more severe, atypical, or clinically notable presentations were preferentially selected for publication, which should be considered when interpreting the pooled frequency and severity estimates presented in this review.

Two further methodological limitations were identified. First, approximately 20% of records had follow-up durations that were either insufficient to capture the full clinical course or inadequately reported, as exemplified by Kanayama (2026) and Khan (2025), potentially underestimating late outcomes including relapse and mortality. Second, several case series, including Cooksley (2025) and Dirven (2023)—did not specify whether reported cases constituted the complete institutional experience or a selected subset, compounding the selection-domain uncertainty noted above and further raising the possibility of preferential reporting of atypical or treatment-refractory presentations. A small subset of studies, notably Dupré (2020) and Yuda (2025), were classified as having moderate methodological concern due to significant gaps in clinical detail or follow-up documentation.

Taken together, ascertainment of exposure, outcome, and follow-up was generally adequate across the included literature, but the pervasive uncertainty in the selection domain is a substantive limitation of the underlying evidence base rather than an isolated concern affecting a minority of studies. This should be borne in mind when interpreting the pooled frequencies of clinical features and outcomes reported in this review, which likely overrepresent severe or clinically notable presentations relative to the true spectrum of ICI-associated HLH.

### Study and Patient Characteristics

A total of 96 patients with ICI-associated HLH/MAS were identified from 78 published reports spanning 2017 to 2026.^5,9-85^ All included evidence was case based: 65 publications were single case reports (83.3%) and 13 were case series (16.7%), contributing 65 (67.7%) and 31 (32.3%) patients, respectively. Cases originated predominantly from the United States, Japan, France, and China, which together accounted for 64 of 96 patients (66.7%). Age data were available for 94 patients, with a median age of 60.0 years (IQR, 42.5–72.0; range, 2–86 years). The cohort comprised 53 males (55.2%) and 43 females (44.8%) (Table 1).

**Table 1.** Study and Patient Characteristics

Characteristic	Pooled result
Included evidence	
Total patients	96
Total reports/studies	78
Country of reported cases (n = 96)	
United States	22/96 (22.9%)
Japan	20/96 (20.8%)
France	11/96 (11.5%)
China	11/96 (11.5%)
United Kingdom	7/96 (7.3%)
Australia	6/96 (6.2%)
Other countries	19/96 (19.8%)
Age, years (n = 94)	
Median age(range)	60.0(2-86)
Sex (n = 96)	
Male	53/96 (55.2%)
Female	43/96 (44.8%)

### Primary Malignancy and ICI Exposure

The most common underlying malignancies were melanoma (26/96, 27.1%) and lung cancer (23/96, 24.0%), followed by breast cancer (9/96, 9.4%) and kidney cancer (8/96, 8.3%). Among the 81 patients with documented disease stage, stage IV disease predominated (59/81, 72.8%), followed by stage III (15/81, 18.5%), stage II (5/81, 6.2%), and stage I (2/81, 2.5%).

PD-1 blockade was the most frequently implicated ICI class (60/96, 62.5%), followed by mixed-class combination regimens, PD-L1 blockade, and CTLA-4 blockade. Overall, 54 patients (56.2%) received ICI monotherapy and 42 (43.8%) received combination therapy. The most commonly reported individual agents were pembrolizumab (36/96, 37.5%), nivolumab plus ipilimumab (19/96, 19.8%), nivolumab (14/96, 14.6%), and atezolizumab (8/96, 8.3%). Among 54 patients with documented treatment line, ICI was administered as first-line therapy in 29 (53.7%). Available timing data indicated that HLH onset occurred early in the treatment course, with a median of 2 cycles prior to HLH diagnosis and a median interval of 30 days from ICI initiation to HLH onset, though both ranges were wide (Table 2).

**Table 2.** Primary Malignancy, Stage, ICI Exposure, and Timing of HLH Onset

Variable	Pooled result
Primary malignancy or underlying disease (n = 96)	
Melanoma	26/96 (27.1%)
Lung cancer	23/96 (24.0%)
Breast cancer	9/96 (9.4%)
Kidney cancer	8/96 (8.3%)
Other malignancies, each ≤3 cases	30/96 (31.3%)
Cancer stage among reported cases (n = 81)	
Stage 4	59/81 (72.8%)
Stage 3	15/81 (18.5%)
Stage 1–2	7/81 (8.6%)
ICI class (n = 96)	
PD-1 blockade	60/96 (62.5%)
Mixed-class ICI regimen	20/96 (20.8%)
PD-L1 blockade	12/96 (12.5%)
CTLA-4 blockade	4/96 (4.2%)
ICI regimen type (n = 96)	
Monotherapy	54/96 (56.2%)
Combination therapy	42/96 (43.8%)
Reported ICI agents (n = 96)	
Pembrolizumab	36/96 (37.5%)
Nivolumab plus ipilimumab	19/96 (19.8%)
Nivolumab	14/96 (14.6%)
Atezolizumab	8/96 (8.3%)
Other reported agents	16/96 (16.7%)
ICI agent not reported	3/96 (3.1%)
Treatment line among reported cases (n =54)	
First line	29/54 (53.7%)
Second line	16/54 (29.6%)
Third line or later	9/54 (16.7%)
Treatment line not reported	42/96 (43.8%)
Timing of HLH onset	
ICI cycles before HLH onset(n=85)	Median 2 cycles; IQR 1–4; range 1–26
Days from ICI initiation to HLH onset (n=65)	Median 30 days; IQR 11–120; range 0–510
Days from last ICI dose to HLH onset (n=67)	Median 14 days; IQR 7–26; range 0–60

### Diagnostic Approach and HLH Features

Diagnostic ascertainment was heterogeneous across publications. Among the 96 patients, a combined approach incorporating both HLH-2004 criteria and HScore was the most frequently employed strategy (31/96, 32.3%), followed by HLH-2004 criteria alone (27/96, 28.1%), HScore alone (23/96, 24.0%), and clinical diagnosis without explicit reference to a formal diagnostic framework (15/96, 15.6%); the latter category likely reflects under-reporting of diagnostic criteria rather than a deliberate methodological choice, and these cases were retained provided they met predefined inclusion standards.

Considering all patients in whom the HScore was reported regardless of the primary diagnostic approach, a numeric HScore was available for 50 of 96 patients, with a median of 233.0. By the published HScore probability calculator, a score of 233 corresponds to a greater than 93% estimated probability of HLH, lending quantitative support to the validity of diagnoses included in this review. HLH-2004 criteria were formally documented in 58 patients (60.4%), with a median of 5 fulfilled criteria among the 47 patients for whom a numeric count was reported; meeting 5 or more of the 8 HLH-2004 criteria is consistent with a formal HLH diagnosis by this tool.

Core HLH features were consistently documented across reported cases. Fever was present in 94 of 96 patients (97.9%), cytopenias in 91 of 93 patients with available data (97.8%), splenomegaly in 52 of 68 (76.5%), hemophagocytosis on bone marrow biopsy in 57 of 76 (75.0%), elevated soluble CD25 in 53 of 55 (96.4%), and reduced or absent NK-cell activity in 16 of 18 (88.9%); the latter finding was subject to considerable reporting limitations. Laboratory parameters were consistent with a severe hyperinflammatory phenotype: median ferritin 10,450 ng/mL, median platelet count 52,000 cells/µL, median triglycerides 298 mg/dL, and median fibrinogen 147 mg/dL (Table 3).

**Table 3.** Diagnostic Ascertainment, HLH-Defining Features, and Key Laboratory Findings

Variable	Pooled result
Diagnostic approach (n = 96)	
Combined diagnostic approach	31/96 (32.3%)
HLH-2004 criteria	27/96 (28.1%)
HScore	23/96 (24.0%)
Clinician diagnosis	15/96 (15.6%)
Diagnostic scores among reported cases	
Numeric HScore available	50/96
HScore	Median 233.0; IQR 201.3–260.3; range 105–319
Fulfilled HLH-2004 criteria available	47/96
Fulfilled HLH-2004 criteria	Median 5; IQR 5–6; range 4–8
Core HLH features	
Fever	94/96 (97.9%)
Cytopenias	91/93 (97.8%)
Splenomegaly	52/68 (76.5%)
Hemophagocytosis in bone marrow	57/76 (75.0%)
Elevated soluble CD25	53/55 (96.4%)
Low or absent NK-cell activity	16/18 (88.9%)
Key laboratory findings	
Ferritin	Median 10,450 ng/mL; IQR 3,931–35,400; n=89
Platelet count	Median 52,000 cells/µL; IQR 32,250–82,000; n=74
Triglycerides	Median 298 mg/dL; IQR 221–355.5; n=63
Fibrinogen	Median 147 mg/dL; IQR 100–250; n=61
AST	Median 195.5 U/L; IQR 83.3–402.3; n=52
ALT	Median 156 U/L; IQR 65.5–335; n=40

### Concurrent Factors

An infectious workup was documented in 84 of 96 patients (87.5%). Epstein-Barr virus (EBV) testing was reported in 46 patients, of whom 9 (19.6%) were positive; cytomegalovirus (CMV) testing was reported in 39 patients, of whom 2 (5.1%) were positive. Additional concurrent infections were identified in 18 of 82 patients with available data (22.0%). Malignancy progression was considered as a potential contributing or confounding factor in 39 patients (40.6%). Concurrent immune-related adverse events (irAEs) were documented in 57 of 96 patients (59.4%) (Table 3).

### Treatment and Supportive Care

**Figure 2.**
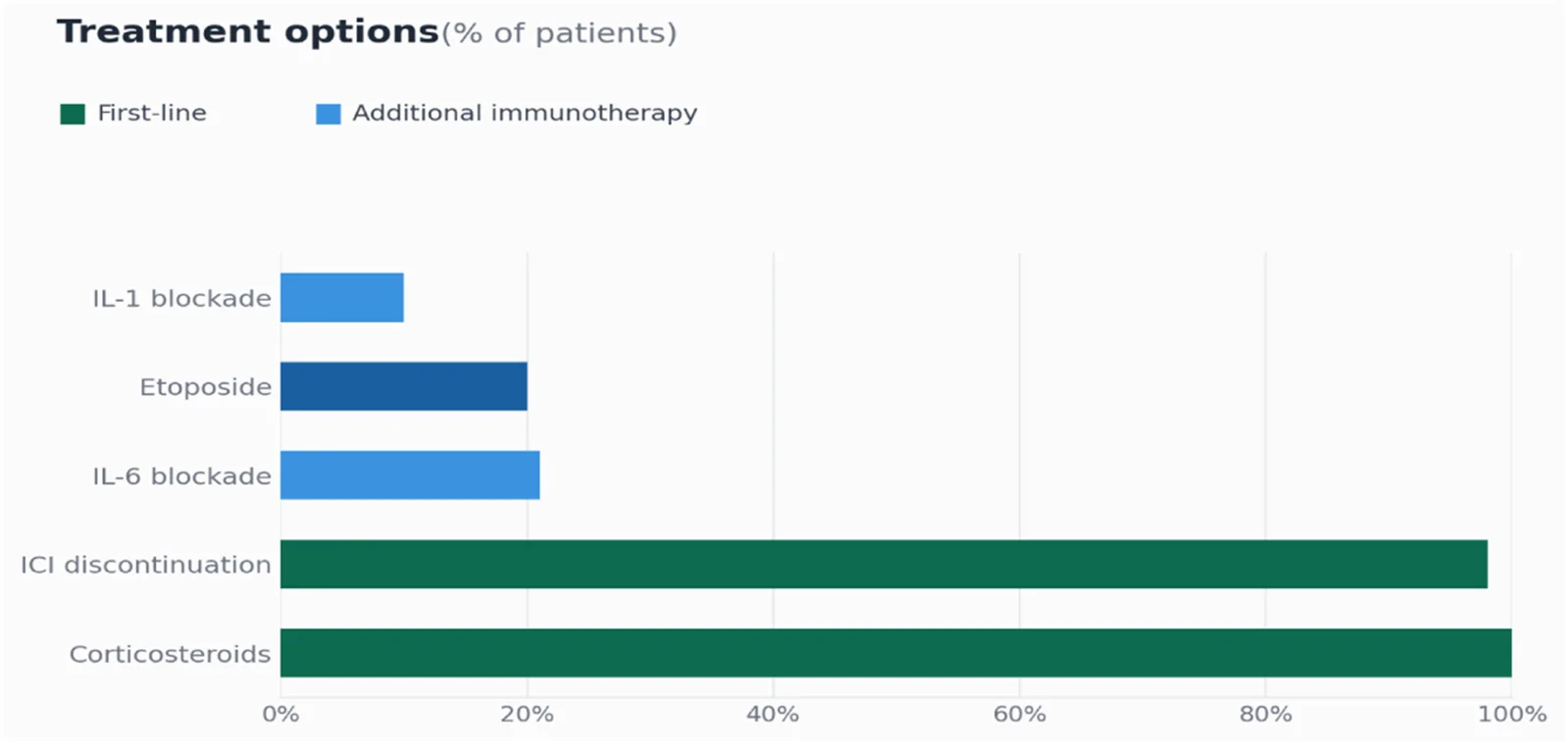
Treatment options used in ICI-associated HLH. Bars represent the percentage of patients receiving each treatment modality. Green bars indicate first-line interventions: corticosteroids (systemic; methylprednisolone, dexamethasone, or prednisone) and ICI discontinuation. Blue bars indicate additional HLH-directed immunotherapy administered beyond corticosteroids. Data derived from 96 patients. ICI, immune checkpoint inhibitor; CS, corticosteroids; IL-6, interleukin-6; IL-1, interleukin-1

ICI discontinuation was reported for 92 patients and was carried out in 90 (97.8%). Systemic corticosteroids were administered in 96of 96 patients (100%); the initial agent was methylprednisolone in 48 patients, dexamethasone in 19, and prednisone in 15, with steroid type unreported in 10 and an alternative or combined regimen recorded in 4. Additional HLH-directed or immunomodulatory therapy was variably employed: IL-6 blockade in 21 patients (21.9%), etoposide in 19 (19.8%), and IL-1 blockade in 10 (10.4%); intravenous immunoglobulin (IVIG) and calcineurin inhibitors were used less frequently (Figure 2).

ICU admission status was documented in 27 patients, of whom 15 (55.6%) were admitted into intensive care units. Organ support data were available for 53 patients; among these, 18 received at least one organ-support modality, including mechanical ventilation, renal replacement therapy, vasopressor support, and other measures (Table 4).

**Table 4.** Treatment, Supportive Care, Clinical Outcomes, and ICI Rechallenge

Variable	Pooled result
Treatment	
ICI discontinued	90/92 (97.8%)
Corticosteroids administered	96/96 (100%)
Initial steroid type	Methylprednisolone 48/96; dexamethasone 19/96; prednisone 15/96
Etoposide administered	19/96 (19.8%)
IL-6 blockade administered	21/96 (21.9%)
IL-1 blockade administered	10/96 (10.4%)
IVIG administered	10/76 (13.2%)
Calcineurin inhibitor administered	6/75 (8.0%)
Supportive care and organ support	
ICU admission among ICU status reported cases	15/27 (55.6%)
At least one organ-support modality	18/53 (34.0%)
Clinical response reported	94/96
Complete response	63/94 (67.0%)
Partial response	15/94 (16.0%)
No response	16/94 (17.0%)
Relapses among reported cases	6/72 (8.3%)
Mortality status reported	95/96
Death	24/95 (25.3%)
Alive at reported follow-up/outcome point	71/95 (74.7%)
Most common recorded causes of death	Cancer progression 9/24; HLH 7/24; infection 3/24
ICI rechallenge performed	7/65
HLH recurrence after rechallenging	3/7
No HLH recurrence after rechallenging	3/7
Rechallenge outcome not reported	1/7

### Clinical Outcomes and ICI Rechallenge

**Figure 3.**
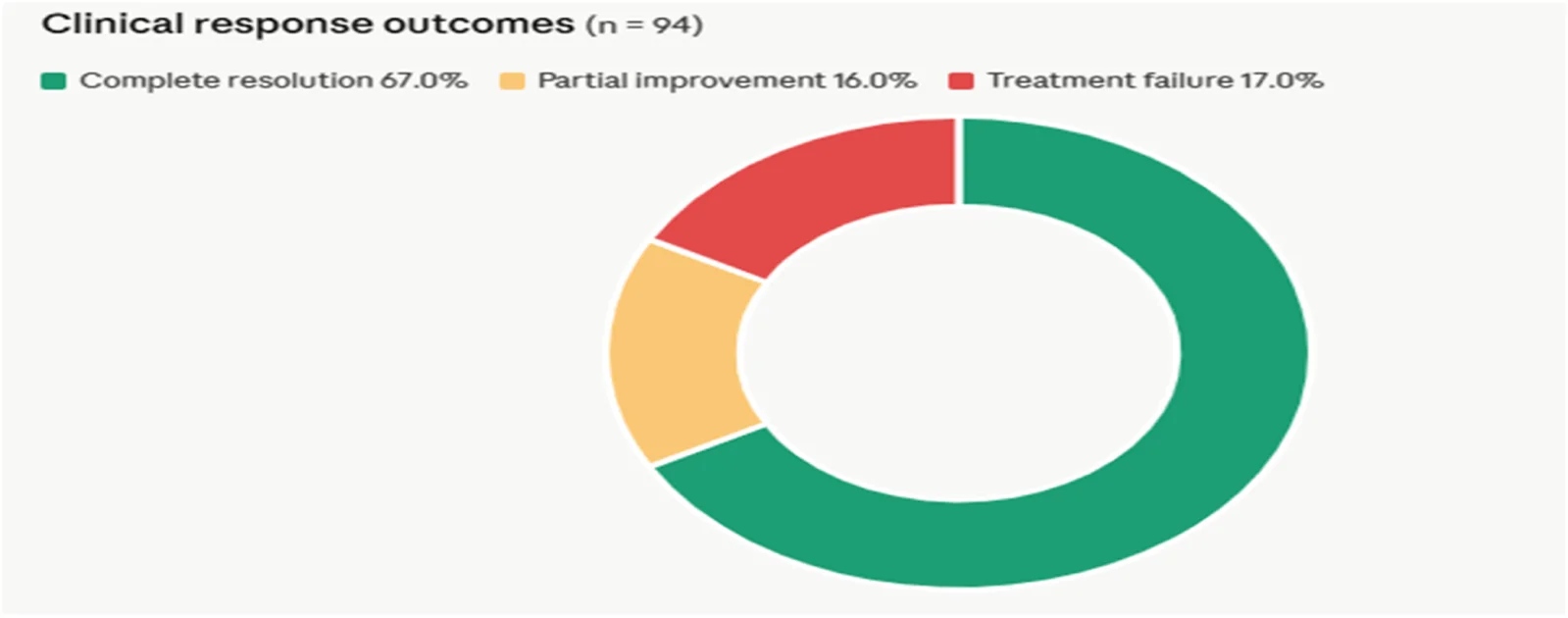
Clinical response outcomes. Graph shows the outcome distribution across 94 patients: nearly two-thirds achieved complete HLH resolution, with only 17% classified as treatment failure. The overall response rate (complete + partial) was 83%

Clinical response was evaluable in 94 patients. Complete HLH resolution was documented in 63 (67.0%), partial improvement in 15 (16.0%), and treatment failure in 16 (17.0%); overall, 78 of 94 patients (83.0%) achieved at least a partial response (Figure 3). HLH relapse was recorded in 6 of 72 patients with applicable follow-up data (8.3%).

Mortality data were available for 95 patients. Death occurred in 24 (25.3%), while 71 patients (74.7%) were alive at the reported follow-up point. Among the 24 deaths, attributable causes were cancer progression (n=9), HLH (n=7), infection (n=3), concurrent HLH and cancer progression (n=2), concurrent HLH and infection (n=1), and other causes (n=2). ICI rechallenge was uncommon and incompletely reported; among 65 patients with available rechallenge data, 7 (10.8%) underwent rechallenge, of whom 3 experienced HLH recurrence, 3 did not, and 1 had an unreported outcome (Table 4).

## Discussion

In this systematic review and pooled patient-level analysis of 96 patients with ICI-associated HLH/MAS drawn from 78 published reports spanning nearly a decade, we provide a comprehensive characterization of the clinical presentation, diagnostic landscape, treatment patterns, and outcomes of this rare but potentially fatal immune-related toxicity. HLH developed early in the treatment course across tumor types and ICI regimens, diagnostic ascertainment was heterogeneous, and mortality reached 25.3% despite near-universal corticosteroid use — collectively highlighting critical gaps in early recognition, standardized diagnosis, and evidence-based treatment escalation.

The observed mortality of 25.3% exceeds 13.7%, 15.3%, and 15% reported in prior reviews by Walmsley et al,^4^ Diaz et al.,^86^ and Zhai et al,^5^ respectively, likely reflecting broader case ascertainment across a longer timeframe that captured a greater proportion of severe and fatal presentations. Our observed mortality rate (25.3%) closely mirrors that reported in the largest available real-world pharmacovigilance dataset on this topic, a FAERS-based analysis of 733 ICI-associated HLH reports by Guzel et al.,^87^ which found a mortality of 25.1%. This convergence, across case-level and large-scale disproportionality data, provides independent external validation of the mortality burden associated with ICI-related HLH and suggests our cohort is broadly representative of the wider reported experience despite its smaller size.

PD-1 blockade was the most frequently implicated class in our cohort (60/96, 62.5%), and combination regimens accounted for 43.8% of cases (42/96) versus 56.2% on monotherapy. Although our case-level design precludes comparative risk estimates, these patterns are consistent with disproportionality signals from Guzel et al, who reported higher reporting odds of HLH with combination therapy—particularly ICI plus targeted agents (ROR 2.17, 95% CI 1.72–2.73)—and with PD-1 relative to PD-L1 inhibitors (ROR 1.86, 95% CI 1.41–2.46).^87^ The two datasets are complementary: ours characterizes clinical phenotype and outcomes at the individual-case level, while the FAERS analysis contextualizes relative reporting risk across regimens at the population level. Notably, 9 of 24 deaths in this cohort were attributed to cancer progression rather than HLH, underscoring that overall mortality figures are subject to attribution bias in patients with concurrent advanced malignancy and should not be equated with HLH-specific case fatality.

ICI-associated HLH is mechanistically rooted in the unchecked activation of cytotoxic T lymphocytes and NK cells following checkpoint disinhibition, leading to IFN-γ–driven macrophage hyperactivation, hemophagocytosis, and the hypercytokinemia responsible for multiorgan failure.^88^ This pathway closely parallels IFN-γ–mediated macrophage activation in CAR-T–associated cytokine release syndrome,^89^ providing biological justification for the use of IL-6 blockade in ICI-HLH — an approach employed in 21.9% of patients in this cohort. The predominance of PD-1–based regimens among reported cases reflects their widespread clinical use and cannot be interpreted as evidence of higher intrinsic biological risk relative to other ICI classes.

HLH-2004 criteria were developed for pediatric primary HLH^2^ and the HScore for a mixed adult reactive HLH population that excluded ICI-treated patients^6^; neither tool was validated in the oncological ICI setting. Key HLH-2004 parameters — particularly NK-cell activity and soluble CD25 — are frequently unavailable in routine practice, while the HScore does not account for the competing causes of fever, cytopenias, and hyperferritinemia that are common in patients with advanced malignancy receiving immunotherapy. Despite these limitations, the median HScore of 233 in this cohort corresponds to a greater than 93% estimated probability of HLH, lending quantitative support to the validity of included diagnoses. Future reports should document both the numeric HScore and the number of fulfilled HLH-2004 criteria; more broadly, development of ICI-specific diagnostic criteria that incorporate the competing etiologies unique to this population is now a research priority.

EBV positivity in approximately 20% of tested patients warrants specific clinical attention. EBV is the most common infectious trigger of secondary HLH,^90^ and ICI-induced immune disinhibition may itself precipitate latent EBV reactivation.^91^ Whether EBV in this context represents an independent trigger, a co-precipitant, or an incidental finding is often indeterminate — yet the distinction matters therapeutically, as etoposide carries efficacy in EBV-HLH through both cytotoxic and indirect antiviral mechanisms.^92^ EBV and CMV testing should therefore be considered mandatory in the diagnostic workup of all patients with suspected ICI-associated HLH. The high prevalence of concurrent irAEs (59.4%), consistent with published data on multi-organ irAE co-occurrence,^93^ raises the possibility that HLH in this context reflects systemic immune dysregulation rather than an isolated hematological toxicity — suggesting that patients with multi-organ irAEs may warrant prospective HLH screening.

An additional consideration relevant to the expanding use of ICIs is the occurrence of HLH in patients receiving these agents with curative intent, in the neoadjuvant or adjuvant setting. Although the majority of reported ICI-associated HLH cases have occurred in the metastatic setting, several cases have now been described in patients treated for early-stage disease, including HLH/cytokine release syndrome following neoadjuvant pembrolizumab-chemotherapy for locally advanced triple-negative breast cancer^46^ and secondary HLH occurring during adjuvant pembrolizumab for triple-negative breast cancer administered per the KEYNOTE-522 regimen.^71^ A further case describes treatment-refractory, ultimately fatal HLH in a patient with resected stage IIC melanoma who had completed four cycles of adjuvant pembrolizumab, complicated by secondary disseminated fungal infection.^81^ These reports underscore that HLH is not confined to the metastatic or advanced-disease population, and that clinicians managing patients on curative-intent ICI regimens must maintain a comparable index of suspicion, since diagnostic delay in this setting carries the same risk of rapid clinical deterioration described elsewhere in this review. As neoadjuvant and adjuvant ICI indications continue to expand across tumor types, prospective pharmacovigilance and future case reporting should specifically capture treatment setting (curative versus palliative intent) to determine whether risk, phenotype, or outcomes of ICI-associated HLH differ meaningfully in this population.

Corticosteroids and ICI discontinuation were near-universal, consistent with ASCO ^94^ and ESMO guidelines ^95^ for severe irAEs. However, neither guideline specifically addresses HLH as a distinct clinical entity, and escalation beyond steroids — employed in a substantial minority with IL-6 blockade (21.9%), etoposide (19.8%), and IL-1 blockade (10.4%) was entirely empirical. Given confounding by indication, no comparative conclusions regarding escalation strategies can be drawn from these data. Facchini et al proposed a severity-adapted management framework for suspected ICI-induced HLH.^96^ In their algorithm, patients should be hospitalized promptly, the ICI should be held, and intravenous methylprednisolone should be initiated early at 1–4 mg/kg/day, with pulse-dose methylprednisolone (up to 1,000 mg/day for 3 days) reserved for critically ill presentations. Clinical and laboratory response should be reassessed within approximately 72 hours; if improvement is observed, corticosteroids can be tapered gradually over 4–6 weeks, whereas steroid-refractory or rapidly progressive cases should prompt escalation to second-line immunomodulation and higher-level care. In this setting, supportive care and multidisciplinary management are emphasized, and options such as anakinra, tocilizumab, IVIG, and, in selected refractory cases, etoposide or other cytotoxic agents may be considered depending on clinical severity and local expertise.

ASCO guidelines advise that rechallenge may be considered once toxicity reverts to grade 1 or below, with grade 4 events generally warranting permanent discontinuation^94^; ESMO similarly recommends individualized benefit-risk assessment for hematological irAEs.^95^ In this cohort, only 7 patients underwent rechallenge, with HLH recurrence in 3, precluding any risk estimation. Broader retrospective data on rechallenge after severe irAEs suggest recurrence risk varies by organ system and severity,^17^ but HLH-specific evidence is absent. Until prospective data exists, rechallenge decisions should be individualized based on cancer status, availability of alternative therapies, completeness of HLH laboratory resolution, and patient preference, and all attempts should be systematically documented.

Several limitations should be acknowledged. First, the exclusive reliance on case reports and case series introduces substantial publication and reporting bias, with severe or atypical cases likely overrepresented, limiting generalizability. Second, mortality (25.3%) and other frequencies are descriptive point estimates from a small, selectively reported population and should not be interpreted as population-level risks. Third, heterogeneous and often limited follow-up may underestimate late relapse or mortality, while cause-specific mortality was based on authors’ attribution rather than independent adjudication. Fourth, treatment comparisons are subject to confounding by indication and cannot establish comparative effectiveness. Finally, heterogeneity in diagnostic criteria, reporting completeness, geographic representation, English-language restriction, and database coverage may further limit generalizability. The review was also retrospectively registered on OSF; although a prior protocol was used, retrospective registration cannot eliminate the potential for selective reporting or methodological bias.

Future work should address three priorities: standardized case reporting inclusive of numeric diagnostic scores, mandatory infectious workup, treatment sequencing, and rechallenge outcomes; prospective denominator-based data capture through pharmacovigilance registries or mandated screening in ICI trials; and mechanistic research into why a minority of ICI-treated patients develop HLH, including germline variants in cytotoxicity pathways, subclinical immune dysregulation, and viral co-triggers, to identify high-risk subgroups and inform biomarker-driven surveillance strategies.

## Conclusion

ICI-associated HLH is a rare but potentially fatal complication of checkpoint immunotherapy, carrying a mortality that exceeds one in four patients despite near-universal immunosuppressive treatment. This systematic review establishes the largest characterization of its clinical phenotype to date, while exposing persistent and consequential gaps in diagnostic standardization, treatment escalation, and rechallenge safety. Prospective registries, ICI-specific diagnostic criteria, and comparative treatment data are now essential prerequisites for meaningful clinical progress in this area.

## Supplemental Material

Supplemental Material - Immune Checkpoint Inhibitor–Associated Hemophagocytic Lymphohistiocytosis: A Systematic Review Supplemental Material for Immune Checkpoint Inhibitor–Associated Hemophagocytic Lymphohistiocytosis: A Systematic Review by Abdulrahman F. Al-Mashdali, Fatihelmgib Mohamed, Marwa Osman, Mujahid O. Abdelraof, Mohammed Abdulgayoom, Shehab F. Mohamed in Clinical Medicine Insights: Oncology.

## Data Availability

All data supporting the findings of this study are included within the article and its supplementary materials. Additional information is available upon reasonable request.*
