# Supplementary material for: Immune Checkpoint Inhibitor–Associated Hemophagocytic Lymphohistiocytosis: A Systematic Review
Source: Clin Med Insights Oncol. 2026 Sep 11;20:11795549261489492. doi: 10.1177/11795549261489492 (PMC13570046; doi:10.1177/11795549261489492)
Supplement: Supplementary file 1 [file sj-pdf-1-onc-10.1177_11795549261489492.pdf]

Table 1. Murad et al assessment of case reports & case series

Study ID	Type of publication	Does the patient(s) represent(s) the whole experience of the investigator (center) or is the selection method unclear to the extent that other patients with similar presentation may not have been reported?	Was the exposure adequately ascertained?	Was the outcome adequately ascertained?	Was follow-up long enough for outcomes to occur?	Is the case(s) described with sufficient details to allow other investigators to replicate the research or to allow practitioners make inferences related to their own practice?
Akagi 2020	Case report	No	Yes	Yes	Yes	Yes
Alan 2025	Case report	No	Yes	Yes	Yes	Yes
Alhouri 2026	Case report	No	Yes	Yes	Unclear	Yes
Ali 2023	Case report	No	Yes	Yes	Yes	Yes
Al-Samkari 2019	Case report	No	Yes	Yes	Yes	Yes
Azari 2021	Case report	No	Yes	Yes	Unclear	Yes
Bertin 2023	Case report	No	Yes	Yes	Yes	Yes
Chen 2024	Case series	Yes	Yes	Yes	Yes	Yes
Chin 2019	Case report	No	Yes	Yes	Yes	Yes
Choi 2021	Case report	No	Yes	Yes	Yes	Yes
Chow 2025	Case report	No	Yes	Yes	Yes	Yes
Cooksley 2025	Case series	Unclear	Yes	Yes	Yes	Yes
Costa 2021	Case report	No	Yes	Yes	Yes	Yes
Dirven 2023	Case series	Unclear	Yes	Yes	Unclear	Yes
Dommaraju 2025	Case report	Unclear	Yes	Yes	Yes	Yes
Doyle 2021	Case report	Unclear	Yes	Yes	Yes	Yes
Du 2022	Case series	Unclear	Yes	Yes	Unclear	Yes

Dupré 2020	Case series	Yes	Yes	Yes	No	No
Endo 2022	Case report	Unclear	Yes	Yes	Yes	Yes
Gajagowni 2025	Case report	Unclear	Yes	Yes	Yes	Yes
Geusens 2024	Case report	Unclear	Yes	Yes	Yes	Yes
Ghous 2021	Case report	Unclear	Yes	Yes	No	Yes
Hagiwara 2024	Case report	Unclear	Yes	Yes	Yes	Yes
Hantel 2018	Case report	Unclear	Yes	Yes	Yes	Yes
He 2023	Case report	Unclear	Yes	Yes	Yes	Yes
Herman 2024	Case report	Unclear	Yes	Yes	No	Yes
Heynemann 2024	Case report	Unclear	Yes	Yes	Unclear	Yes
Hidaka 2024	Case report	Unclear	Yes	Yes	Unclear	Yes
Holmes 2022	Case report	Unclear	Yes	Yes	Yes	Yes
Honda 2025	Case report	Unclear	Yes	Yes	Yes	Yes
Honjo 2019	Case report	Unclear	Yes	Yes	Yes	Yes
Jiao 2025	Case report	Unclear	Yes	Yes	Yes	Yes
Kalmuk 2020	Case report	Unclear	Yes	Yes	Yes	Yes
Kanayama 2026	Case report	Unclear	Yes	Yes	No	Yes
Kawamura 2025	Case report	Unclear	Yes	Yes	Yes	Yes
Khan 2025	Case series	No	Yes	Yes	No	Yes
Krasovitsky 2021	Case report	No	Yes	Yes	Yes	Yes
Kurozumi 2021	Case series	Unclear	Yes	Yes	Unclear	Yes

Laderian 2019	Case report	Unclear	Yes	Yes	Unclear	Unclear
Long 2024	Case report	No	Yes	Yes	Yes	Yes
Lorenz 2019	Case report	No	Yes	Yes	Yes	Yes
Malissen 2017	Case series	Unclear	Yes	Yes	Yes	No
Marar 2022	Case report	No	Yes	Yes	Yes	Yes
Masood 2021	Case report	No	Yes	Yes	No	Yes
Michot 2018	Case report	Yes	Yes	Yes	Yes	No
Mosalem 2024	Case report	No	Yes	Yes	Yes	Yes
Nelson 2022	Case series	Yes	Yes	Yes	Yes	Yes
Okawa 2019	Case report	Unclear	Yes	Yes	Unclear	Yes
Olivares-Hernández 2021	Case report	No	Yes	Yes	Unclear	Yes
Olmes 2025	Case report	No	Yes	Yes	No	Yes
Ota 2024	Case report	Yes	Yes	Yes	Yes	Yes
Oyama 2023	Case report	No	Yes	Yes	Yes	Yes
Ozdemir 2020	Case series	Unclear	Yes	Yes	Unclear	Yes
Pacholczak-Madej 2022	Case report	No	Yes	Yes	No	Yes
Patton 2024	Case report	Unclear	Yes	Yes	No	Yes
Peres 2025	Case report	No	Yes	Yes	Yes	Yes
Pinto Valdivia 2025	Case report	No	Yes	Yes	Yes	Yes
Ramos-Ruperto 2021	Case report	No	Yes	Yes	Unclear	Yes
Rollet 2024	Case report	No	Yes	Yes	Unclear	Yes

Rubio-Perez 2022	Case report	Unclear	Yes	Yes	Yes	Yes
Sadaat 2018	Case report	Unclear	Yes	Yes	Yes	Yes
Sakai 2020	Case report	Unclear	Yes	Yes	No	Yes
Salazar-Zuniga 2025	Case report	Unclear	Yes	Yes	Unclear	Yes
Sasaki 2018	Case report	No	Yes	Yes	Yes	Yes
Satzger 2018	Case report	Unclear	Yes	Yes	Yes	Yes
Shah 2017	Case report	Unclear	Yes	Yes	No	Yes
Shi 2026	Case report	No	Yes	Yes	Yes	Yes
Shibata 2026	Case report	Yes	Yes	Yes	Unclear	Yes
Takahashi 2020	Case report	Unclear	Yes	Yes	No	Yes
Takeshita 2017	Case report	No	Yes	Yes	Yes	Yes
Thummalapalli 2020	Case report	Unclear	Yes	Yes	Yes	Yes
Tiu 2021	Case series	Yes	Yes	Yes	Unclear	Yes
Virdi 2025	Case report	No	Yes	Yes	No	Yes
Walmsley 2024	Case report	Unclear	Yes	Yes	Yes	Yes
Wei 2022	Case series	Unclear	Yes	Yes	Yes	Yes
Wei 2024	Case report	Yes	Yes	Yes	Yes	Yes
Yuda 2025	Case series	Yes	Yes	Unclear	Yes	Unclear
Zhai 2024	Case report	Yes	Yes	Yes	Yes	Yes

Murad et al. Checklist. The Murad et al. checklist was used to appraise the methodological quality of case reports and case series. The tool evaluates four key domains: representativeness of the reported case(s), adequacy of exposure ascertainment, adequacy of outcome ascertainment, and sufficiency of follow-up and reporting detail to allow replication or clinical inference. Each domain is rated as Yes, No, or Unclear; no composite numerical score is generated. Studies were considered suitable for inclusion when the majority of domains were adequately addressed.
